# Clinical Effectiveness and Cost-Effectiveness of Oral-Health Promotion in Dental Caries Prevention among Children: Systematic Review and Meta-Analysis

**DOI:** 10.3390/ijerph16152668

**Published:** 2019-07-25

**Authors:** Nadine Fraihat, Saba Madae’en, Zsuzsa Bencze, Adrienn Herczeg, Orsolya Varga

**Affiliations:** 1 Department of Preventive Medicine, Faculty of Public Health, University of Debrecen, H-4002 Debrecen, Hungary; 2 Department of Clinical Pharmacy, Faculty of Pharmacy, University of Jordan, Amman 11942, Jordan

**Keywords:** Oral Health Promotion Programs (OHPP), Decayed Missing Filled Teeth (DMFT), cost-effectiveness analysis (CEA), Incremental Cost Effectiveness Ratio (ICER)

## Abstract

The objective of this study was to evaluate the clinical effectiveness and cost-effectiveness of oral-health promotion programs (OHPPs) aiming to improve children’s knowledge of favorable oral health behavior to lower decayed/-missing/-filled teeth (DMFT) while reducing the financial cost on health institutions. An electronic search was performed in seven databases. Studies were restricted to human interventions published in English. The search study followed the Preferred Reporting Items for Systematic Reviews and Meta-Analysis (PRISMA) guidelines, and the risk of bias was assessed based on the Drummonds Checklist. A total of 1072 references were found. Among these, 19 full texts were included. Most studies had a strong quality. The overall pooled impact of OHPPs estimates children suffering from DMFT/S to have 81% lower odds of participating in OHPP (95% CI 61–90%, I^2^: 98.3%, *p* = 0). Furthermore, the program was shown to be effective at lowering the cost in 97 out of 100 OHPPs (95% CI 89–99%, I^2^: 99%, *p* = 0). Three subgroups analyses (age groups, study countries, studies of the last five years) were performed to evaluate the influence modification on the pooled effect. A comprehensive analysis of the OHPPs confirmed a reduction effect on child DMFT, hence, lowering the financial burden of dental-care treatment on health institutions.

## 1. Introduction

Dental caries represents a globally known preventable non-communicable diseases which is considered a major public-health problem affecting all age groups, especially children. Health promotion that goes beyond health care puts health on the agenda of policymakers in order to achieve better health outcomes [1]. Oral health promotion plays an essential part in the health of the general promotion [2] since the inter-relationship between oral and general health has been approved [1], for instance, through strong statistical correlation between periodontitis and diabetes [3]. Thus, oral- and general-health promotion addresses the inseparable issues of all systemic and oral-health-diseases, specifically through general and oral hygiene, general- and oral- health-care attitudes, and general-health and dental-care services [2]. In fact, dental programs and oral health prevention programs rarely receive the same level of attention as medical care among decision-makers when taking into account the cost-effective allocation of scarce health care resources [4]. However, to allocate scarce healthcare resources, further information and studies are needed based on health economic evaluation [5]. Moreover, considering the economic impact of dental caries on different populations in countries around the world would serve health authorities for reaching reliable public-health decisions regarding the cost of oral-health diseases.

In the context of oral health, however, the 2016 Global Burden of Disease Study “estimated that oral diseases globally affect at least 3.58 billion people, with caries of permanent teeth, being the most prevalent of all assessed conditions. Globally, it is estimated that 2.4 billion people suffer from caries of permanent teeth, and 486 million children suffer from caries of primary teeth” [6]. Dental caries is the most prevalent chronic disease among children, and dental care is the greatest unmet healthcare need [7]. According to the World Health Organization (WHO), in European countries tooth decay among six-year-old children varies from 20% to 90% [8]. Approximately, a quarter of five- to six-year-old children experience tooth decay, and the percentage rises above 90% in some low- and middle-income countries, indicating dental caries is a permanent public-health problem [9]. WHO oral-health goals have been formulated for the year 2020 as part of the WHO Health 21 policy for Europe [10] suggesting that “by 2020, a percentage of at least “80%” of children at the age six should be caries-free and, on average, no more than 1.5 Decayed/-Missing/-Filled Teeth should be observed for children of 12 years of age”.

Given the extent of the problem, the economic burden of dental caries treatment is a large share of many countries’ healthcare budget. Consequently, a study in Colombia measured the economic impact of dental caries 2011, where the cost of dental caries represented 0.02% of 2011 at the current GDP, which means that there was approximately an expenditure of $1.46 for each Colombian citizen to treat dental caries, where the government could draft cost-effective oral-health policies to reduce dental caries prevalence in Colombia’s population [11]. In order to estimate dental caries expenses among children, the Medical Expenditure Panel Survey reported in 2006 that approximately 19% of children younger than 5 years old had dental expenditures of $729 million [12].

Since education and oral -health prevention programs for all family members, children and parents, at all socio-economic levels are the only means to avoid dental caries [8], dentists and oral healthcare providers prioritize oral-health promotion [8]. To achieve such goals, Oral Health Promotion Programs (OHPPs) for children are globally implemented in diverse communities and have been shown to be a useful intervention to control dental caries. However, economic evaluation of their cost-effectiveness to determine the programs value for money remains unclear.

This review seeks to determine if implementing an appropriate oral -health promotion program reduces dental caries among children, and the financial cost on healthcare institutions. We hypothesize that exposure to oral health promotion programs reduce dental caries among children, and health care costs.

## 2. Materials and Methods

The review protocol was registered in the international database of prospectively registered systematic reviews in health and social care (PROSPERO), Centre for Reviews and Dissemination, University of York (No: CRD 42019125611). Although there is no standard protocol for economic evaluation studies, one of the reviews in the literature recommended a protocol to improve the preparation of reviews of healthcare economic evaluation [13]. The criteria for considering studies for this review are as follows:

### 2.1. Type of Studies

The review included trial and model-based economic evaluation studies.

### 2.2. Included Participant, Intervention, Comparator, and Outcome (PICO) Terms

Participants:

- Children aged from 0 to 12 years old who were healthy without health-related diseases except for dental caries.
- Studies of mixed populations of parents and children were included where the data of children were presented separately.

Interventions:

- Community-based oral-health education/training programs related to healthy oral habits.
- Screening of children’s teeth.
- Supervised toothbrushing technique through the provision of toothbrushes, an appropriate amount of fluoride toothpaste, and topical fluoride.
- Advice on dietary control, such as limitation of sugar or carbohydrates consumption, and enhanced fortified nutrition with an appropriate amount of calcium intake.

Comparator: not providing an oral-health promotion program or could have been providing a differing action than the intervention group, within similar conditions.

Context: OHPPs implemented by oral-health professionals in the contexts of home visits, telephone calls, healthcare centers and primary schools.

Outcomes:

- Reducing the “Decayed, Missing, Filled Teeth (DMFT) Index for permanent teeth or (DMFT) Index for deciduous teeth” among children.
- OHPP cost, incremental cost (difference between mean costs of intervention and mean costs of the comparator), and cost-effectiveness analysis (CEA).

The search strategy and selection process included relevant PICO terms, prospectively defined, following the Preferred Reporting Items for Systematic Reviews and Meta-Analysis (PRISMA) guidelines. The initiated search date was 11 December 2018, where the restriction concerning the publications was for the English language. The following bibliographic databases were searched, and Mesh terms and Emtree were used: PubMed: “ “Costs and Cost Analysis”(Mesh) AND (“Oral Health”(Mesh) OR “Dental Caries”(Mesh) OR “Dental Care for Children”(Mesh)) AND (“Child”(Mesh) OR “Child, Preschool”(Mesh))”; Excerpta Medica Database (EMBASE): “(‘mouth hygiene’/exp OR ‘dental caries’/exp OR ‘dental prevention’/exp OR ‘dental procedure’/exp) AND ‘economic evaluation’/exp AND ‘child’/exp”; DARE, NHSEED and HTA: (Oral Health OR Dental Caries OR Dental Care) AND (Child OR Preschool Child OR Infant); Cost-Effectiveness Analysis (CEA) Registry: “Dental Caries, Oral Health, Dental care”, Paediatric Economic Database Evaluation (PEDE): “Dental Caries”.

The title, abstract and full text of each study were screened and accurately assessed. The used method for the selection criteria was importing the searched outcomes in the bibliographic reference software EndNote X7 to remove duplicate records and to precisely screen records through two phase screening. During the first phase screening of titles and abstracts, irrelevant records were basically categorized as intervention, opinion, reviews, participants, outcomes, not English language, the exclusion criteria of irrelevant records were clearly explained as participant with health-related diseases or aged older than 12 years; interventions other than OHPP, such as implant dentistry or other invasive-dentistry programs; other economic-evaluation outcomes such as cost-benefit, cost-utility or cost-minimization; authors’ opinion (unoriginal records); study reviews; and study language other than English. The second-phase screening completely assessed the full article text of articles to verify the level of consistency that the studies had with the eligibility criteria. In addition, data extraction was gathered by formulating two tables using Windows Excel 2013 (Microsoft, Redmond, WA, USA), to separately collect the qualitative and quantitative data. (Appendix A, Table A1 and Table A2)

Meanwhile, data that were extracted from the included criteria studies the risk of bias in studies that were detected simultaneously. The Drummond Checklist provides useful guidance applied to clarify the included studies with 10 answerable questions (yes, no, or not available), assuming the assessment result as strong, moderate, or weak, (see Table A3 and Table A4) [14].

Table A3: represent 10 trial-based economic evaluation studies assessed by 10 questions of the Drummond checklist.

Table A4: represent nine model-based economic evaluation studies assessed similarly by nine questions of the Drummond checklist.

For meta-analysis, we included eight studies; the missing data dealt with contacting study authors. STATA Software version 14 (StataCorp LP., College Station, TX, USA) was used. Where the pooled figures were multiplied by 100 due to software technical competency, a few missing data were replaced by the number 1 as an integer. The cost in diverse countries with different currencies was converted to 2015 prices of USA dollars. For studies using the USA dollar, we measured the inflation rate for each study considering the 2015 standard year. Data analysis was performed through founded dichotomous outcomes such as the number of children in the intervention and in the control group, the DMFT index in children, and the OHPP cost. Odds ratio (OR) is an effect size with 95% confidence interval (CI) and study weights were estimated from random effects analysis. Forest plots for each needed outcome were demonstrated, and the chi-square test was used to assess whether the observed differences were homogeneous or heterogeneous where a *P* value of less than 0.1 indicated statistically significant heterogeneity. An I^2^ test was used to quantify inconsistencies between studies as the percentage of variation across studies was measured where heterogeneity was quantified as 0% to 40% implying slight heterogeneity, 30% to 60% implied moderate heterogeneity, 50% to 90% implied substantial heterogeneity and 75% to 100% implied very substantial (considerable) heterogeneity. Data synthesis was carried out using narrative demonstration, with a summary of the characteristics of each included study. For quantitative synthesis, a summary of the combined estimation related to the OHPP effect was measured. Due to heterogeneity analysis, three subgroups were performed to assess the modification influence on the pooled effect through the age of the children, studies of the last 5 years and the country of the study. Egger’s regression test and a funnel plot were used to assess and demonstrate publication bias, as publication bias was considered present if the *p*-value of the Egger test was more than 0.05.

## 3. Results

**Figure 1:**
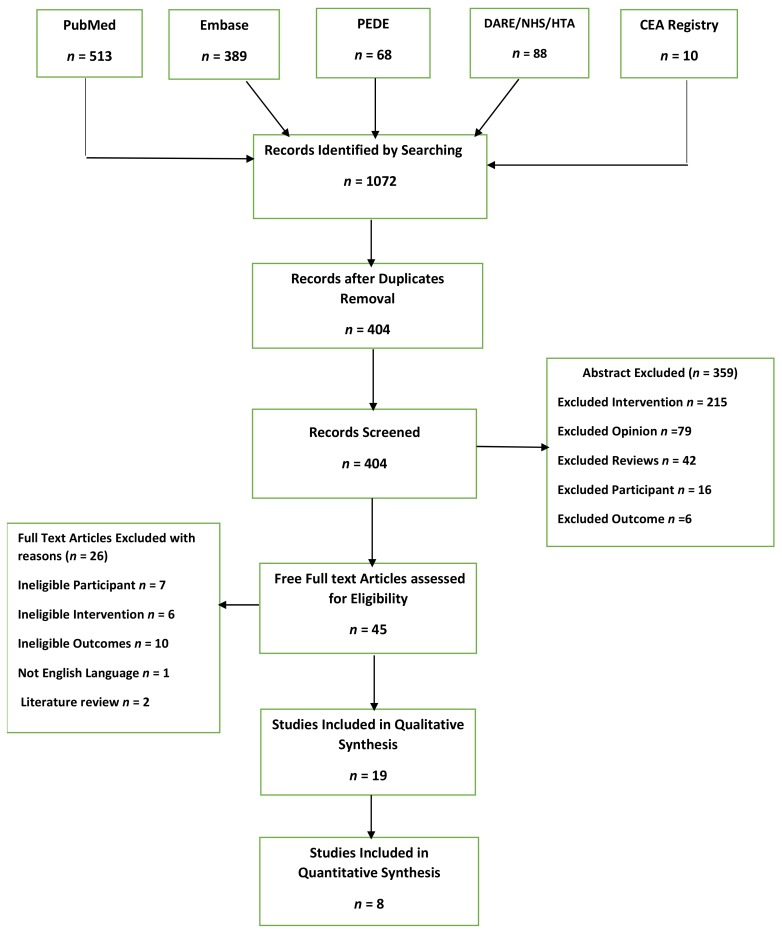
Preferred Reporting Items for Systematic Reviews and Meta-Analysis (PRISMA) flow diagram for the inclusion of studies.

Overall, 1072 records were retrieved for eligibility screening. After removal of duplicates, 404 records were obtained. Screening of titles and abstracts excluded 359 records, given the proper reasons when records were not relevant to the aim of the review. We assessed 45 full texts of articles and identified 19 articles for qualitative synthesis and eight articles for quantitative synthesis. Figure 1 shows the PRISMA flow diagram for the inclusion of studies.

The 19 included studies [5,15,16,17,18,19,20,21,22,23,24,25,26,27,28,29,30,31,32], which are illustrated through the general characteristics, in Table A1 in Appendix A had one or more intervention(s) in the included OHPPs, while the majority of studies examined the implementation of OHPPs in the intervention group compared with dissimilar or absent intervention in the control group. 

Furthermore, the included studies originated from diverse countries: United Kingdom (n = 6; 31.6%), Australia (*n* = 5; 26%), United States (*n* = 3; 16%), Finland (*n* = 1; 5%), Japan (*n* = 1; 5%), Nigeria (*n* = 1; 5%), Singapore (*n* = 1; 5%) and Ireland (*n* = 1; 5%) with diverse currencies used in different time longevity of the OHPPs. In addition, the review is based on (*n* = 9; 47%) trial-based economic-evaluation studies, and (*n* = 10; 52%) model-based economic evaluation studies with two different age groups where 14 studies were of younger than 6-year-old children and four studies age were of older than 6 years old, and one study did not mention the age of the children. Of all studies, 47% were published in the last 5 years.

For the quantitative data, Table A2: presents the cost-effectiveness outcomes of the included studies. Table A2: presents the cost in the intervention and in the control group, measured by the incremental cost based on the type of found outcomes in the included studies, such as DMFT, Average of dental visits, number of prevented caries teeth, average of cavity-free months, probability of less cost, caries percentages, number of into the mouth of babes program visits (no. of IMB), quality-adjusted life year (QALY) (measure of disease burden, including the quality and the quantity of life lived, it can be used in cost-effectiveness studies to assess the value for money of clinical interventions), cost-effectiveness ratio, and percentages of not having debris. The table also includes the outcome-effect result in the intervention and the control groups, Incremental Cost Effectiveness Ratios (ICERs), cost saving, indirect cost, and total program cost.

Nineteen studies were included in the systematic review and meta-analysis, and the Drummond Checklist [14] was used to assess the risk of bias in the trial- and model-based economic evaluation studies. The included studies were ten trial-based economic evaluation studies and nine model-based economic evaluation studies. Out of the 19 included studies, 12 studies had a low risk of bias and seven had a moderate risk of bias.

### 3.1. According to the Review, to Summarize the Studies That Met the Inclusion Criteria

#### 3.1.1. Strong-Quality “Model-Based” Economic Evaluation Studies

A study by Kowash [15], which was conducted in the UK over three years, aimed to provide a dental health education program of home visits with mothers of eight-month-old young infants to prevent early-childhood caries (ECC). It provides strong evidence for the reductions of dental caries associated with deemed cost saving based on program intervention; DMFT had a 0.29 score in the intervention group, and 1.75 in the comparative group. The author concluded that “oral health-education gave better costs-effectiveness ratios than another preventive program”. In addition, a study by Pukallus [16] estimated that oral-health advice of oral health therapists, delivered within five and a half years through calling the parents with children with a mean age of 1 year, saved $108,406.92 the year of 2012.

The 2006 study by Quinonez [17] aimed to examine the cost-effectiveness of fluoride varnish application by medical providers. The intervention was the application of universal fluoride varnish at 9, 18, 24, and 36 months as cycles extended to 42 months. The study analyzed cavity-free months, which were equal to 31.49 in the intervention group and 29.97 in the control group; in terms of cost, intervention cost was $181.66 in 2003 and $170.73 in the control group in the same year; hence, it increased incremental cost to $10.93 in 2003.

Furthermore, Stearns [18] estimated the cost-effectiveness of medical office-based preventive oral health, where the program was effective in the terms of cost. The cost of intervention was $54.81 which is less than the cost of control $285.8 as cost-saving reached $33.64 in 2006.

A study by Anopa [19] which was about a national supervised tooth-brushing program, found the program to save cost. It aimed to compare the cost of providing a supervised toothbrushing program with National Health Service (NHS) cost savings. It assumed the total reduction in tooth decay in five-year-old children was due to the tooth brushing program. The study measured the avoided cost per DMFT as incremental cost about (−) $197.44 in 2009, and the cost-saving was estimated to be $6,912,617 within the same time frame. Thus, the authors speculated that the cost-savings of the tooth brushing program can be successful in most socio-economically deprived children.

Another study by Blaikie [20] was preliminary economic analysis that was conducted in Australia over seven years from 1970 until 1976 to study the cost of school dental care for school-age children to provide the best care at the lowest cost. It compared the fee-for-services-based program with the regular community dental health branch cost in which the founded costs were $3,259,846 for the free for service an oral screening program and $3,034,576 for community dental health branch in the year of 1976. The author suggested that “the Dental Health Branch was more cost-effective than the proposed fee-for-service alternative as the program is an economically acceptable method of delivering school dental care”.

Moreover, Samnaliev [21] was entitled to measure the cost-effectiveness of a disease management program for early childhood caries. The caries percentages in the case group were equal to 4.15% compared with the control group which was equal to 22.5%, thus for the incremental cost of the program was equal (−) $8380 in the year of 2011, where the cost-saving reached $904 in the same year. It appeared that “the program is cost-effective and has the potential to reduce healthcare costs”. 

#### 3.1.2. Moderate Quality “Model-Based” Economic Evaluation Studies

Takeuchi’s [22] study was conducted in Japan for 12-year-old children; the study proved its health effect to decrease DMFT score in the intervention group as it was scored (2.2) DMFT compared with (4.86) DMFT score in the control group. It could not prove the effect in the terms of cost when the total program cost reached $2432.52 in 2006. The second moderate quality study was by Plonka [23]; a longitudinal study of home visits compared to telephone contacts to prevent ECC. The program was effective in terms of health gain, as the caries percentages in the case group reached 2% compared with the control group, which was 15%. The study did not take into account the cost-saving of the program, hence the author concluded: “the home visits and telephone contacts conducted every 6 months from time of birth are effective in reducing ECC prevalence by 24 months”.

#### 3.1.3. Strong-Quality “Trial-Based” Economic Evaluation Studies

Tickle [24] measured the effects and costs of a dental caries prevention regime for young children. Although the intervention group had lower DMFT than the control group, the intervention program cost was higher, with incremental costs reaching $167.61 in 2015. Additionally, Donaldson [25], based in the UK, gave additional information about the three-year-long study of caries reduction after topical application of 4% sodium fluoride per oral (NaFPO). Although it reduced caries, the program incremental costs reached $71.97 in 1974. 

In contrast, an Irish study by O’Neill [26] took three years, in which participants were centrally randomized into the intervention of 22,600 ppm fluoride varnish, toothbrush, a 50-mL tube of 1450 ppm fluoride toothpaste, and standardized prevention advice, while the control group with oral health advice only. Although the program was effective in reducing caries, incremental cost in 2014 was $350.06. For older children aged 11–12, a strong-quality study by Hietasalo [5] assessed the cost-effectiveness of a preventive program including a package of oral health advice, preventive treatment, and free materials that were delivered by dental hygienists for 497 children with at least one active caries lesion. The author estimated an incremental cost per Decayed Missing Filled Surface (DMFS) avoided $87.78 in 2004. 

#### 3.1.4. Moderat Quality “Trial-Based” Economic Evaluation Studies

Reiss [27] encouraged the low-income families of 51 children to seek dental care for their children. The incremental cost in 1976 reached $27.68. Koh [28] conducted a study over five and a half years that evaluated the cost-effectiveness of home visits and telephone contact in preventing ECC in children aged from six months to six years. The perspective of the analysis was societal, considering the costs to the parent and the health system. Where the program was effective in terms of gained QALY, intervention cost reached $68 in 2014 compared with the control cost which reached $8448 in the same year. Incremental cost reached (−) $8380 in 2014. Another trial, reported by Davies [29] evaluated the cost-effectiveness of a postal toothpaste program to prevent caries in five-year-old children. The report found that free toothpaste on four occasions a year, and a toothbrush once a year for four years, was effective to reduce the DMFT to a score 2.15 in the intervention group, compared with the control group, with a DMFT score of 2.57. Cost-saving reached $2217.45 in 1992. Economic analysis resulted in an overestimation of the cost and underestimation of the benefits.

#### 3.1.5. Limited Economic-Evaluation Outcomes of Moderate Qualified “Trial-Based’ Economic-Evaluation Studies and “Model-Based” Economic-Evaluation Studies”

Although the three remaining studies (Folayan [30], Lai [31], and Gibbs, L [32]), delivered reliable OHPPs, the economic evaluation of the program could not be demonstrated as needed. Folayan [30] aimed to determine the association between the use of recommended oral self-care caries (ROSC) prevention tools and the presence of dental caries in children residing in suburban Nigeria. The intervention group were encouraged to brush more than once a day, use fluoridated toothpaste, and to eat sugary snacks between main meals less than once a day. The study intervention used ROSC caries prevention tools in combination. Conversely, the control group was exclusively using ROSC prevention tools. It was found that the use of the combination of ROSC caries prevention tools made the probability less costly and more efficient, as the probability of less cost in the intervention group was 98.6%, while it was 61.5% in the comparative group. 

Lai [31] aimed to examine the clinical efficacy of a two-year oral health program for infants and toddlers. The intervention group undertook oral-health education on tooth brushing and fluoride use, non-nutritional habits, trauma prevention, and use of topical fluoride varnish, and this was compared with no oral-health education. Consequently, mean caries reached seven in the intervention group, whereas, it reached 20 in the control group. However, the odds of severe ECC in the control group were three times higher than the intervention group.

A study by Gibbs [32] was based on child oral-health promotion, enrolling migrant families in Australia. The community oral-health education sessions were led by peer educators. Follow-up health messages were given in the intervention group, and the control group had no oral health education. The percentage of not having debris was estimated to be 56% higher in the intervention than the controlled group. The author concluded that intervention of oral-health education session was likely to improve knowledge, behavioral skills, and also adherence to following up. The program cost was $362,329.66 in 2012.

### 3.2. Meta-Analysis

**Figure 2:**
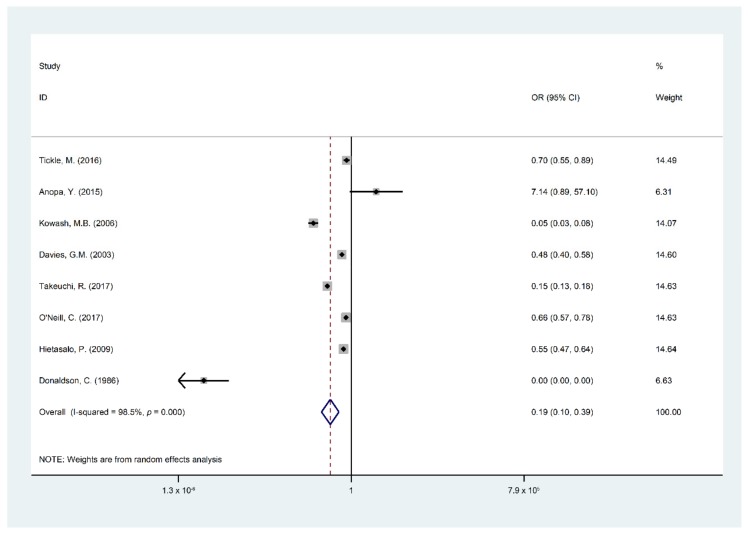
Forest plots of Decayed Missing Filled Teeth (DMFT)/S by the participating children.

**Figure 3:**
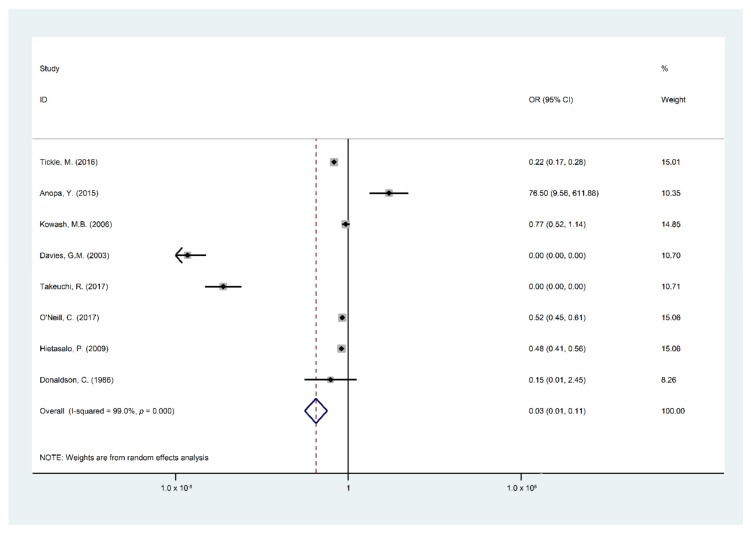
Forest plot of incremental cost-effectiveness per DMFT/S.

**Figure 4:**
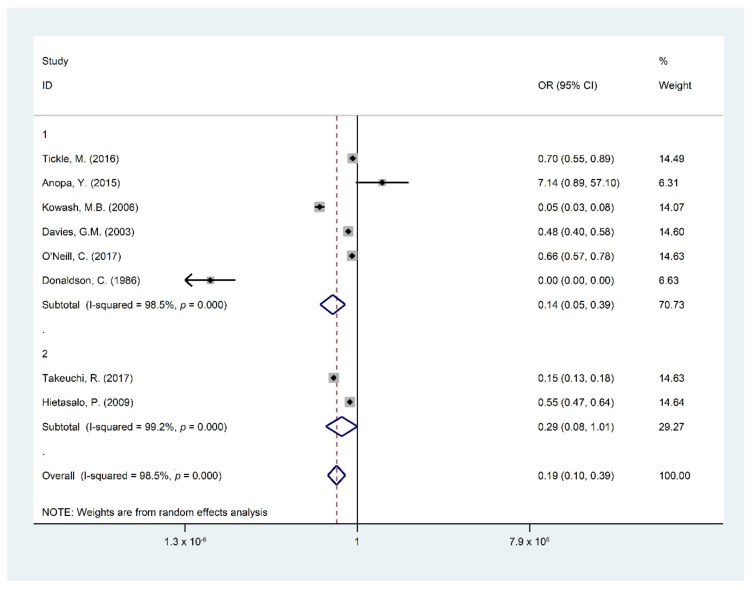
Forest plot of DMFT/S by children age group: 1 as (Age > 6) and 2 as (Age ≤ 6).

**Figure 5:**
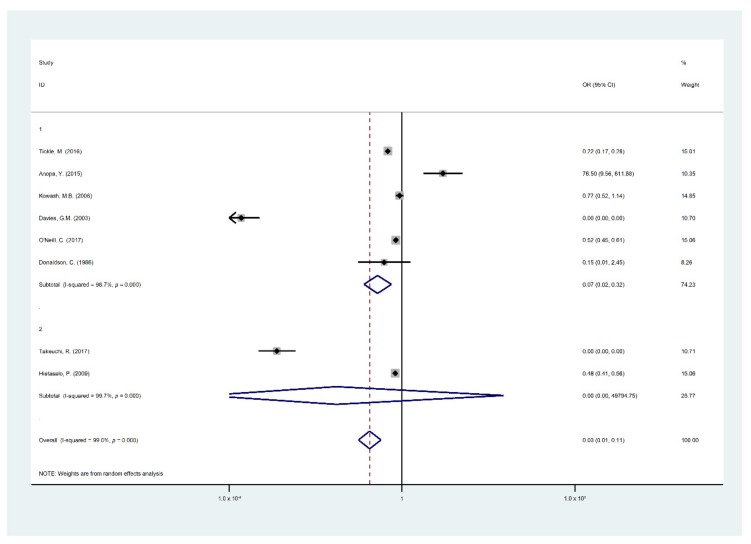
Forest plot of the incremental cost-effectiveness of the intervention and the control groups by the age groups: 1 as (Age > 6) and 2 as (Age ≤ 6).

**Figure 6:**
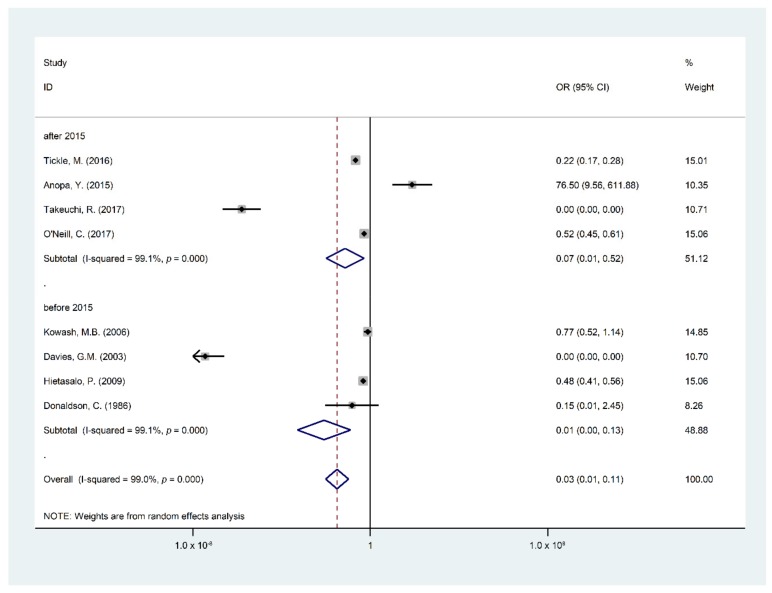
Forest plot for the difference in the DMFT of the intervention group compared to the control group regarding the study years.

**Figure A1:**
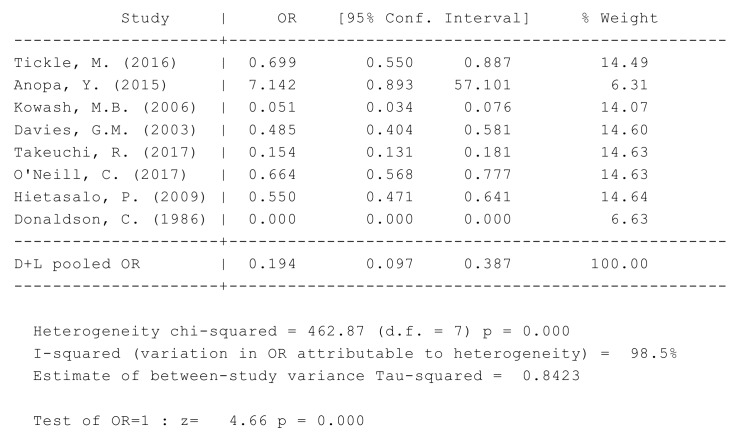
STATA do-files for analysis of Figure 2. *“metan DMFTintervention NIntervention, DMFTcontrol Ncontrol, label (namevar = Author, yearvar = year) random or”*.

**Figure A2:**
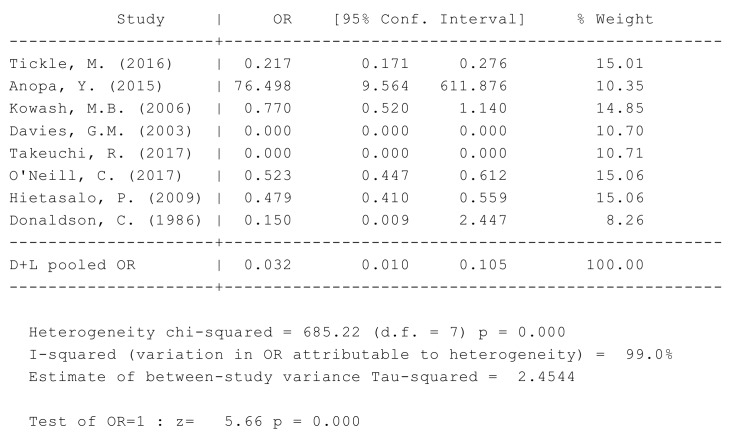
STATA do-file for analysis of Figure 3. “metan DMFTintervention Costintervetion DMFTcontrol Costcontrol, label (namevar = Author, yearvar = year) random or”.

**Figure A3:**
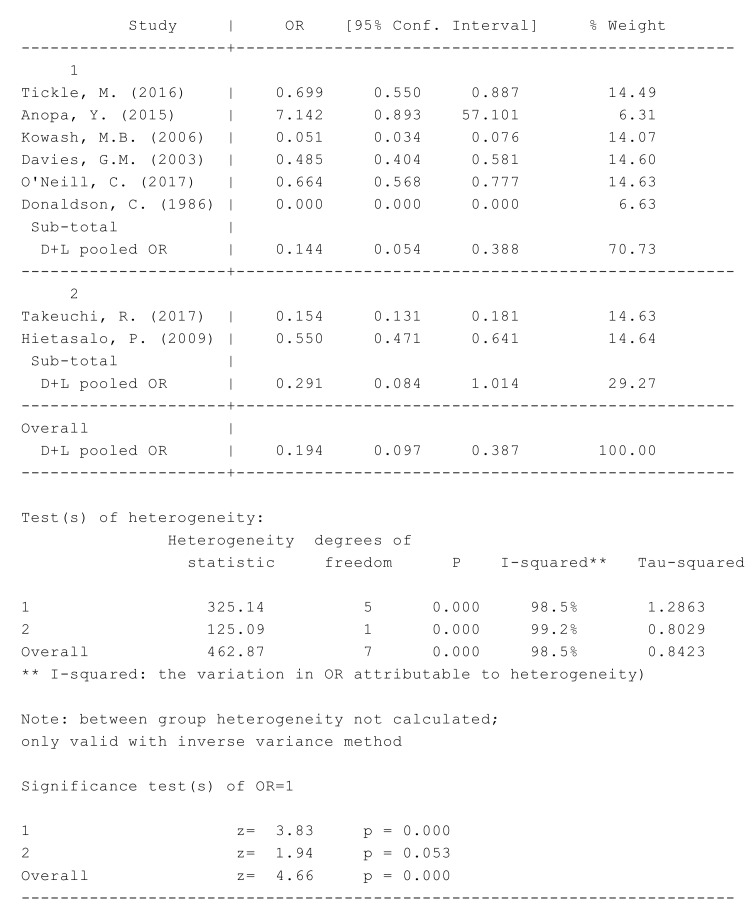
STATA do-file for analysis of Figure 4. *“metan DMFTintervention NIntervention DMFTcontrol Ncontrol, label (namevar = Author, yearvar = year) by (age) random or”*.

**Figure A4:**
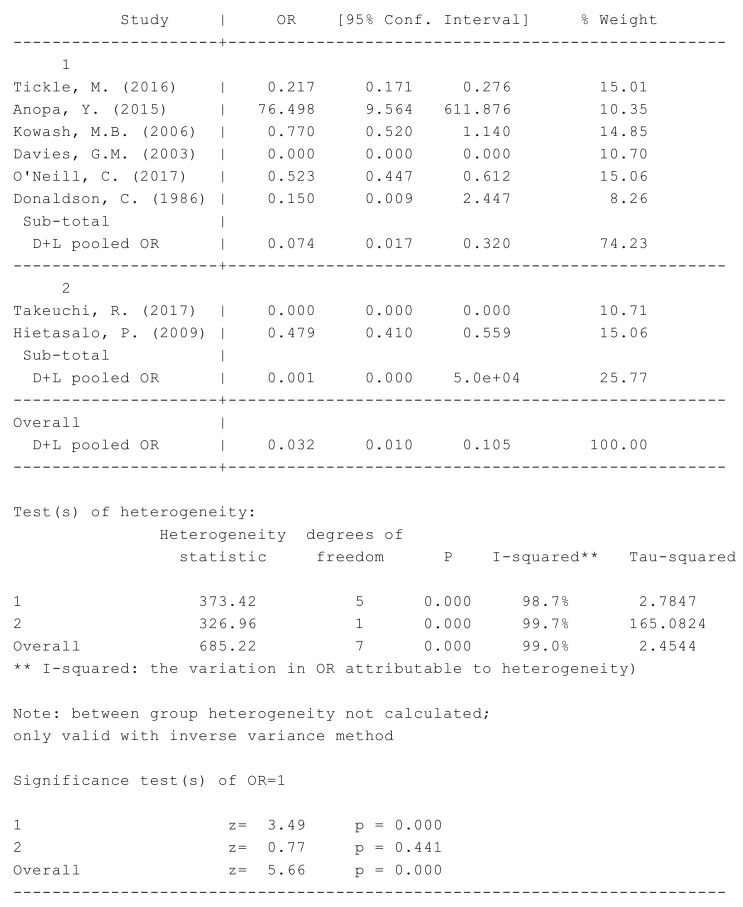
STATA do-file for analysis Figure 5. *“metan DMFTintervention Costintervetion DMFTcontrol Costcontrol, label (namevar = Author, yearvar = year) by (age) random or”*.

**Figure A5:**
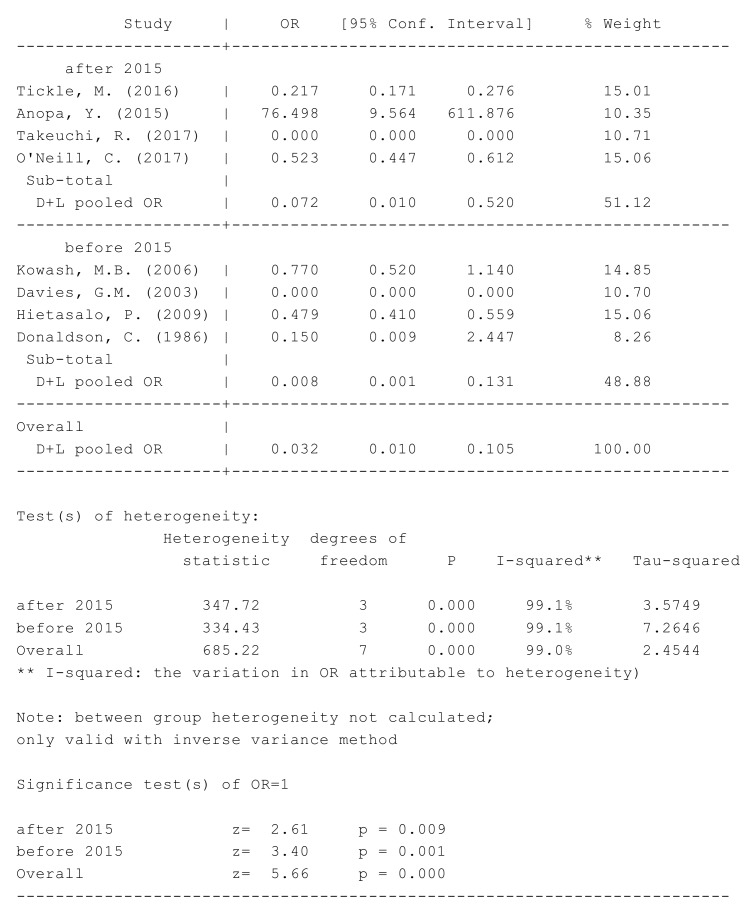
STATA do-files for analysis of Figure 6. *“metan DMFTintervention Costintervetion, DMFTcontrol Costcontrol, label (namevar = Author, yearvar = year) by (One less than 2015 two 2015 onwards) random or”*.

**Figure A6:**
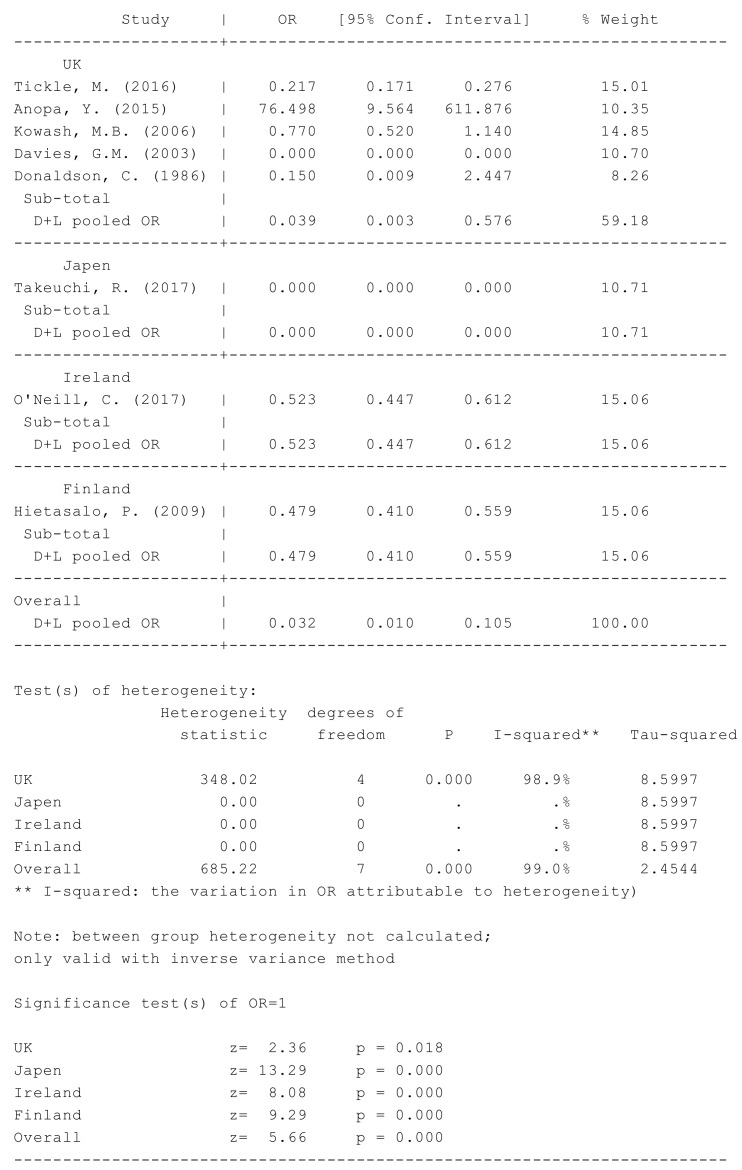
STATA do-files for analysis of Figure 7. *“metan DMFTintervention Costintervetion, DMFTcontrol Costcontrol label (namevar = Author, yearvar = year) by (Country) random or”*.

Findings of the meta-analysis summarized through eight studies [5,15,19,22,24,25,26,29] were included in the quantitative analysis of the effect and cost effect of the OHPPs. The eight selected studies for meta-analysis were analyzed based on the incremental cost of the OHPPs per DMFT and Decayed Filled Teeth (DFT) or DMFS. The cost-effectiveness outcomes are presented in Table A2, covering incremental cost, type of study outcomes, ICER, cost saving, indirect cost, and total program cost. Major findings of the meta-analysis are presented in Figure 2, Figure 3, Figure 4, Figure 5 and Figure 6, STATA do-files for analysis of the figures are presented in the (Appendix B, Figure A1, Figure A2, Figure A3, Figure A4, Figure A5 and Figure A6.

Figure 2 shows that the overall pooled impact of OHPP estimates in children who suffer from DMFT/S had 81% lower odds to participate in OHPP (95% CI 61–90%, I^2^: 98.5%, *p* = 0) with considerable heterogeneity among studies. The reference categories were used in the measurement: “DMFT/S in the intervention group and the number of children in the intervention group”, “DMFT/S in the control group and the number of children in the control group”.

Figure 3 illustrates that the OHPPs had a successful intervention in reducing financial costs in 97 out of 100 OHPPs (95% CI 89–99%, I^2^: 99%, *p* = 0) with considerable heterogeneity among studies. The reference categories were used in the measurement “the cost of the program in the intervention group and DMFT/S in the intervention group”, and “the cost of the program in the control group and DMFT/S in the control group”. 

Due to considerable heterogeneity between the included studies, subgroup analysis was measured to assess the influence modification on the pooled effect by children’s age groups, study countries, and publication date before and after 2015. Figure 4 represents the subgroup analysis according to the age groups.

Studies reported children of less than six years weighted 70.73% with an OR of 0.14 (95% CI, 0.05–0.39, I^2^: 98.5%) had the highest benefit of OHPPs to lower DMFT/S, while studies reporting children aged six years and older weighted 29.27% with an OR of 0.29 (95% CI, 0.08–1.01, I^2^: 99.2% ) had no benefit from OHPPs in lowering DMFT/S. Reference category measurements were: “DMFT/S in the intervention group and the number of children in the intervention group”, and “DMFT/S in the control group and the number of children in the control group” by two age categories.

Figure 5 shows studies that reported children who were less than six years old with an OR of 0.07 (95% CI, 0.02–0.32) revealing no cost-effectiveness effect to reduce OHPP incremental cost, whereas studies reporting children aged six years and older with an OR of 0.0 (95% CI, 0.00–48,704.6) was cost-effective in reducing the OHPPs incremental cost in this age group. The reference category measurements were “DMFT/S in the intervention group and the cost of the program in the intervention group”, “DMFT/S in the control group, the cost of the program in the control group)” by the two age categories.

Studies published after 2015 weighted 51.13% revealed a clinical effect of OHPPs to reduce DMFT as an OR of 0.08 (95% CI 0.01–0.53); studies published before 2015 weighted 48.87% revealed a significant effect of OHPPs to reduce DMFT among children with an OR 0.01 (95% CI 0.00–0.13). The reference category measurements were “DMFT/S in the intervention group and number of children in the intervention group”, “DMFT/S in the control group and the number of children in the control group” by two group study year publishment.

**Figure 7:**
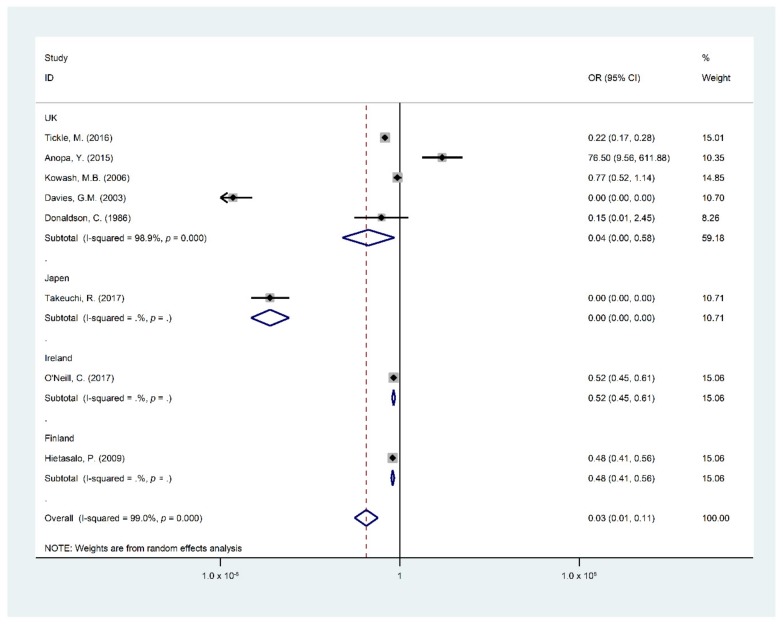
Forest plot for the difference in the DMFT/S of the intervention group compared to the control group by study countries.

Moreover, the study countries of the OHPPs were analyzed, (see Figure 7). The United Kingdom country weighted 59.18%, revealed significant proof that OHPPs had a reducing effect on DMFT/S as an OR of 0.04 (95% CI 0–0.58). The same findings were seen in Japan, Ireland, and Finland, countries with an overall OR of 0.03 (95% CI 0.01–0.11) resulting as “OR 0 (95% CI, 0–0) weighted 10.71%, OR 0.52 (95%CI, 0.45–0.61) weighted 15.06%, and OR 0.48 (95% CI 0.41–0.56) weighted 15.06%” respectively. These countries had significant impact on the overall pooled effect to prove that OHPPs had a reduction effect of DMFT/S among children. The measured reference categories were “DMFT/S in the intervention group and number of children in the intervention group”, “DMFT/S in the control group and the number of children in the control group” by the study countries.

**Figure 8:**
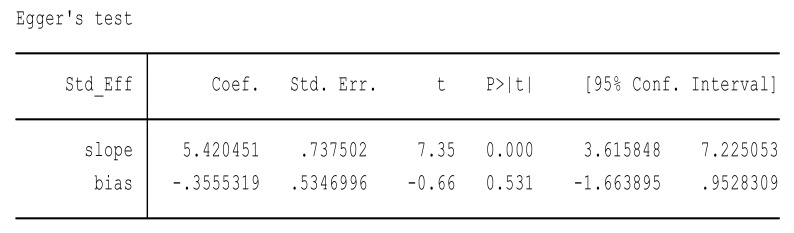
Eggers regression test to test hypothesis 1.

**Figure 9:**
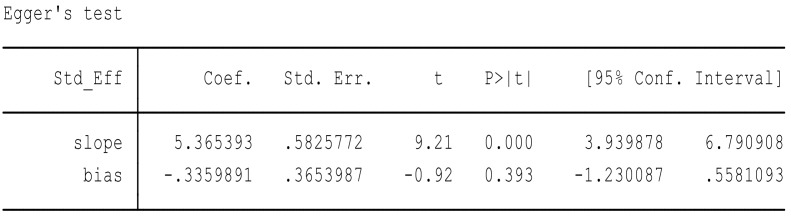
Eggers regression test to test hypothesis 2.

Due to considerable heterogeneity, the Eggers regression test was performed to analyze for publication bias. A publication bias is considered present if the *p*-value of the Egger test is more than 0.05. Major findings of the Eggers regression tests are presented in Figure 8 and Figure 9.

In this way, we assumed two hypotheses. H null: the result of meta-analysis had no effect of DMFT among children with no small sample size; and H alternative: the result of meta-analysis had a reducing effect of DMFT among children with a small sample size. A *p*-value of 0.53 was more than 0.05 in the review and publication bias was present; thus, we reject the null hypothesis and accept the alternative hypothesis.

We assumed two hypotheses. H null: the result of meta-analysis had no effect of OHPP to reduce the financial cost on health institutions with no small sample size; and H alternative: the result of the meta-analysis had a reducing effect of OHPP on the financial cost on health institutions with a small sample size. A *p*-value of 0.39 was more than 0.05 in the review and publication bias was present; thus, we reject the null hypothesis and accept the alternative hypothesis.

**Figure 10:**
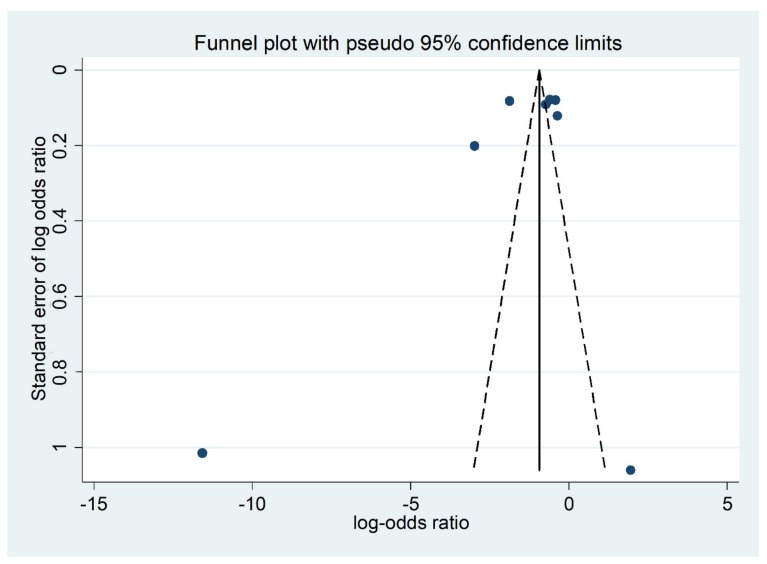
Funnel Plot represented from the eight pooled trial and model-based economic-evaluation studies of Oral Health Promotion Programs (OHPPs), with log-odds ratios displayed on the horizontal axis and the standard error of the log-odds ratios displayed on the vertical axis.

We used the “funnel plot” tool to demonstrate the reason for the publication bias in the meta-analysis. It is a simple scatter plot of the treatment effects, as the ratio measures the odds ratio plotted on a log scale estimated from individual studies (horizontal axis) against a measure of the study size (vertical axis), (see Figure 10).

Figure 10 illustrates the asymmetrical scatter plot of the program effects estimated from the selected individual studies against a measure of the small study size. The funnel plot is seen in this review as the tendency for the smaller studies in a meta-analysis to show a larger treatment effect, where publication bias is only one of a number of possible causes of funnel plot asymmetry [33]. Heterogeneity exists in this review for many reasons. We can explain that the review included diverse studies with various risks in the control group and effect size differs according to study size, intensity of intervention, differences in underlying risk and data irregularities between the pooled studies.

## 4. Discussion

The main review objectives were to assess the clinical effectiveness of oral-health promotion programs on the oral health of children, specifically dental caries, as well as the cost-effectiveness of the programs. The review included 19 studies reporting the used OHPPs and the incremental costs within a year’s related costs. The studies were timely diverse publication between 1976 and 2018, with long-term perspectives.

As stated in 1986 by the WHO in the Ottawa Charter for Health Promotion, the aim was “to enable people to increase control over and to improve their health”. Tooth decay is a preventable and controllable disease. Health promotion improves the quality of life, but it requires a commitment to practicing healthier behavior. Subsequently, it can only be achieved when oral-health promotion activities are implemented at the community level. 

OHPPs in our review mainly implemented dental-health education, focusing on supervised toothbrushing techniques, using the appropriate type of fluoride toothpaste, following healthy behavior, avoiding unhealthy dietary habits and performing regular dental checkups. Where the included criteria studies were conducted within a time frame that varies from one study to another, some studies agree that the longer the time frame of OHPPs the more effective they are in manifesting a favorable oral health result in children’s teeth. However, most health economic studies in this field are not extensive enough to capture most cost- and clinical- outcome differences between the different programs and interventions.

Our review highlights the limited number of economic studies evaluating one type of dental caries prevention intervention, OHPPs, especially with regards to cost-saving, and how the OHPPs could be cost-effective when tooth decay can be avoided. Contemporary evidence shows that decisions on public health policy, health insurance, and client treatment should incorporate economic factors of health expenses. Health economic research studies are able to answer the practical question of “which intervention gives the greatest benefit proportional to its cost?”, while methods to estimate the economic impact of oral diseases are limited in availability, with no harmonized international reporting standards, resulting in difficulty to estimate the full economic impact of oral diseases [34]. 

This review is a reliable indication of the clinical effectiveness and cost-effectiveness of oral-health community programs among children. In a comprehensive way, trial and model-based economic-evaluation studies proved that OHPPs were significantly related to the anticipated review hypothesis regarding the efficacy of dental-health status and financial cost. However, it seems to be inconsequential in terms of OHPP efficacy when comparing two age groups. For clarity, the review found that OHPPs can be clinically effective under the age of six years but in comparison, the program is costly and needs further fiscal management by health authorities. OHPPs, on the other hand, do not reveal higher clinical effectiveness in dental caries reduction among children aged older than six years but they show that the program could not be that much costlier. In the last five years, published studies have also approved the efficacy of OHPPs. Similarly, previous studies represented significantly positive findings of the program, as well as when comparing between several countries. Five studies from the United Kingdom revealed that OHPPs have a significant effect in improving dental health status and reducing the cost of healthcare systems; other countries, namely, Japan, Ireland and Finland, identified the significant effect of the review objectives. Therefore, OHPPs revealed their effects on children’s oral health, on parental dental-treatment expenses, on the health care institutions and on the countries’ GDP. The review findings minimize the knowledge gap between evidence-based research and clinical practice since the program proves the applicability of health promotion in oral healthcare.

### Study Limitations

Although the review included timely diverse studies over seventy years that provide an impressive result in the era of dentistry, the economic evaluation of OHPPs fluctuated based on the year to which the program cost is related, offering a slight chance to generalize the pooled findings. On the other hand, the 19 reviewed studies estimated the paid resources cost when OHPPs commenced from comparative health economic studies, where the counted numbers of studies prove the incremental cost is higher when oral-health promotion taking place in local community health programs. In addition, cost-effectiveness is determined in some studies in terms of tooth decay, before applying intervention that resulted in weakness in the generalizability of cost-effectiveness studies in the area of dental care.

The review protocol included settings and interventions that varied, such as providing oral healthcare, dental screening, and the clinical assessment of children’s tooth health status, applying topical fluoride varnishes, and offering oral health care products (for instance, fluoridated toothpaste and toothbrushes) might be expected to confound the estimated effects of oral-health education. Accordingly, economic studies are limited in terms of generalizability of cost-effectiveness related to specific oral-health intervention due to the differential costs between countries. For example, the cost of a dental filling in one country is more expensive than other countries. Moreover, it is not clear how much a national health service is willing to pay per avoided DMFT/S. It is doubtful to interpret the cost-effectiveness findings in terms of dental caries in the absence of valuable distinct indicators.

## 5. Conclusions

More effort is needed to manage the allocation of scarce resources, taking into account the economic impact of dental caries on healthcare systems. Additionally, more studies are needed regarding caries-prevention methods among young age children in high-, middle- and low-income countries, with follow-up programs to analyze clinical and financial efficacy when conducting well-organized oral-health interventions.

## Appendix A

**Table A1:** The general characteristics of the included studies.

S.no	Lead Author	Year	Country	Study Design	Participant	Mean Age (Year)	Intervention	Main Conclusion	Source of Funding	Quality Assessment
1.	Hietasalo, P. [5]	2009	Finland	Trial-Based	497 children who had at least one active initial caries lesion at baseline of the study	11.5	1. Designed a centered regimen for caries Control 2. Fluoride Varnish	The experimental regimen would be more cost-effective than standard care if the follow-up the period had been longer	Finnish Dental Society Apollonia, the Yrjo Jahnsson Foundation	Strong Quality
2.	Kowash, M.B. [15]	2006	United Kingdom	Model-Based	7000 infants aged 8 months	0.6666	Long-term dental health education program through home visits	Dental Health Education program of home visits with mothers of young infants to prevent early childhood caries gave better benefit-costs and cost-effectiveness ratios than other preventive programs.	British National Health Service (UK) fees	Strong Quality
3.	Pukallus, M. [16]	2013	Australia	Model-Based	Mothers in the intervention group were telephoned when their children were aged approximately 6, 12 and 18 months	1	A telephone Oral Health prevention program	A telephone intervention likely to generate considerable benefits and cost savings to the public dental health service in disadvantaged communities	Australian Centre for Health Services Innovation	Strong Quality
4.	Anopa, Y. [19]	2015	United Kingdom	Model-Based	Hypothetical cohorts of 1000 children aged 5 years	5	Nursery tooth brushing program	Tooth brushing program represents a preventative spend of both reduced costs and health gains in child oral health outcomes.	E-Government through NHS payments	Strong Quality
5.	Blaikie, D.C. [20]	1977	Australia	Model-Based	Community School Children	NA	Free for an Oral screening program compared with Regular community dental health branch	Dental Health Branch was more cost-effective than the proposed fee-for-service alternative.	Department of Public Health ledger listings	Strong Quality
6.	Tickle, M. [24]	2016	United Kingdom	Trial-Based	Children aged 2–3 years, who were caries free at baseline	3.1	1. Oral Health advice 2. providing Toothbrushes and Toothpaste 3. Flouride Varnish	The intervention was unlikely to be cost-effective in terms of either keeping children caries free.	The National Institute for Health Research (NIHR) Health Technology Assessment program	Strong Quality
7.	Quinonez, R.B [17]	2006	United Kingdom	Model-Based	Application of universal fluoride varnish at 9, 18, 24, and 36 months the cycles extended to 42 months to account for benefits incurred after the last Fluoride varnish application at the 36-month well-child visit.	2.1250	Application of fluoride varnish at different times.	Fluoride varnish used in the medical setting is effective in reducing ECC in low-income populations but is not cost saving in the first 42 months of life.	Supported by grant (R01DE013949) National Institute of Dental and Craniofacial Research	Strong Quality
8.	Reiss, M.L. [27]	1976	United States of America	Trial-Based	51 children who needed immediate dental care (determined by dental screening at a local school).	4	1. Oral Health Note. 2. Telephone Contact, Home Visit Oral Health education	The 3 Prompt and 1 Prompt plus 5 Incentive was significantly more effective in initiating dental visits than the Note-Only procedure	Not Reported	Moderate Quality
9.	Donaldson, C. [25]	1986	United Kingdom	Trial-Based	161 children who entered the program and attended continuously for a period of 4 years.	7	Personal health education, oral fluoride supplements applications of acid phosphate fluoride gel and pit and fissure sealing.	There is a need for further study measuring dental outcome which combine aspects of both the quality and length of life of teeth.	Chief Scientist Office of the Scottish Home and Health Department.	Strong Quality
10.	O’Neill, C. [26]	2017	Ireland	Trial-Based	1096 children aged 2 to 3 year attending general practice assigned in 2-arm parallel group to measure the cost-effectiveness of caries prevention program	2.5	1. Fluoride varnish 2. Toothbrush 3. Oral health advice	This trial raises concerns about the cost-effectiveness of a fluoride-based intervention delivered at the practice level in the context of a state-funded dental service	A state-funded dental service	Strong Quality
11.	Koh, R. [28]	2015	Australia	Trial-Based	296 Children aged 6–60 months. 188 home visit interventions; 58 telephone contact interventions; 40 reference controls: usual home care.	3.25	A home visit relative to a telephone call Oral Health advises	Both the home visits and telephone calls were highly cost-effective than no intervention in preventing early childhood caries	National Health and Medical Research Council of Australia	Moderate Quality
12.	Samnaliev, M. [21]	2015	United States of America	Model-Based	518 Children younger than 60 months with active caries or a history of caries	2.5	Oral Disease management program	The program appears cost-effective and has the potential to reduce health care costs	Health care costs were obtained from the hospital finance department. And non-health care costs were estimated through a parent survey	Strong Quality
13.	Plonka, K.A. [23]	2013	Australia	Model-Based	325 children were recruited from community health centers, randomly assigned to receive either a home visit or telephone call.	0.1150	Oral Health education by the home visit and Telephone call.	Home visits and telephone contacts conducted every 6 months from birth are effective in reducing ECC prevalence by 24 months.	The Dental Board of Queensland and the following Queensland Health Departments	Moderate Quality
14.	Stearns, S.C. [18]	2012	United States of America	Model-Based	209,285 Medicaid enrolled children at age 6 months.	3.25	1. Screening and risk assessment 2. Parental counseling, topical fluoride. 3. Topical fluoride application.	The program is cost-effective with 95% certainty if Medicaid is willing to pay 2331 per hospital episode avoided.	Lead Author is independent of any commercial funder	Strong Quality
15.	Takeuchi, R. [22]	2017	Japan	Model-Based	Tongan schoolchildren	12	1. Enforcement of lectures application of fluoride. 2. Instructions on toothbrushing Oral health education. 3. Application of fluoride	The materials for fluoride mouth rinsing and Tooth brushes are lower than for the treatment of caries.	These activities were supported by the JICA	Moderate Quality
16.	Davies, G.M [29]	2003	United Kingdom	Trial-Based	A cohort of children aged 12 months was recruited from a high caries risk population in 9 health districts.	3	Children received toothpaste 1450 ppm fluoride	The program achieved a significant caries reduction in children who received 1450 fluoride toothpaste.	Not Reported	Moderate Quality
17.	Folayan, M.O. [30]	2016	Nigeria	Model-Based	Children living with their biological parents or legal guardians	6.5	Dental health education program of home visits	The use of a combination of fluoridated toothpaste and twice-daily tooth brushing had the largest effect on reducing the chance for caries in children resident in Ile-Ife, Nigeria.	Not Reported	Moderate Quality
18.	Lai, B. [31]	2018	Singapore	Trial-Based	90 children and their caregivers participated in the program, and 64 children were recruited as the control group.	2	Oral program includes tooth brushing, fluoride use and topical fluoride varnish	The odds of severe early childhood caries in the control group were 3 times higher than that for the intervention group	Not Reported	Moderate Quality
19.	Gibbs, L. [32]	2015	Australia	Trial-Based	Families with 1–4-year-old children, 197children in the intervention group and 144 children in the control group Residing in Melbourne.	2.5	1. Community education sessions 2. Follow-up health messages	The Teeth Tales intervention was promising in terms of improving oral hygiene and parent knowledge of tooth brushing technique	Australian Research Council Linkage grant	Moderate Quality

NHS = National Health Service. ECC = early-childhood caries.

**Table A2:** Cost-Effectiveness Outcomes of the standardized included studies.

Author	Economic Study Design	Year to Which Costs Applied	Currency Used to Which Cost Applied	Outcome: Cost-Effectiveness of the Standardized Year 2015 and USD Currency									
				Cost of Study Intervention	Cost of Study Control	Incremental Cost	Type of the Outcomes	Effect of Intervention	Effect of Control	|ICER|	Cost Saving	Indirect Cost	Total Program Cost
Tickle, M. [24]	Trial-Based	2015	£	$242.76	$75.15	$167.61	DMFT	1.15	1.64	342.06	NA	$1341.93	$2872.75
Anopa, Y. [19]	Model Based	2009	£	$24.6	$235.23	(−) $210.63	DMFT	0.08332	NA	1621.7	$737,453.43	NA	$274,762.01
Koh R. [28]	Trial-Based	2013	$	$354,983.72	$185,039.65	(−) $169,944.07	QALY	540	547	24,277.7	$317,174.06	$2197.64	$747,775.07
Reiss, M.L. [27]	Trial-Based	1976	$	$180.95	$65.65	$115.30	Dental Visits	0.846483	NA	32.7	$208.28	$122.76	$66.19
Kowash, M.B. [15]	Model Based	1995	£	$10,046.06	$46,670.13	(−) $36.63	DMFT	0.29	1.75	25,085	$56,716.19	NA	$20,093.67
Pukallus, M. [16]	Model Based	2012	£	$31,059.39	$140,146.01	(−) $109,086.63	No. of caries teeth prevented	11	54	2537	$109,086.63	NA	$31,059.39
Quinonez, R.B. [17]	Model Based	2003	$	$234	$219.92	$14.08	cavity free months	31.49	29.97	9.26	NA	NA	$3816.75
Davies, G.M. [29]	Trial-Based	1992	£	$232,664.49	NA	NA	DMFT	2.15	2.57	61.728	$1845.89	$16,111.88	$755,737.89
Hietasalo, P. [5]	Trial-Based	2004	€	$602.74	$518.36	$84.38	DMFS	2.56	4.6	41.363	$48.56	NA	$278,717.29
Takeuchi, R. [22]	Model Based	2006	$	$2806.96	NA	NA	DMFT	2.2	4.86	NA	NA	$52.91	$2859.86
Folayan, M.O. [30]	Model Based	2015	₦	NA	NA	NA	Probability of less cost	98.60%	61.50%	NA	NA	NA	NA
Samnaliev, M. [21]	Model Based	2011	$	$71.65	$8901.61	(−) $8829.96	Caries%	4.15%	22.50%	48,119	$952.54	$120.73	$8969.92
Plonka, K.A. [23]	Model Based	NA	$	NA	NA	NA	Caries%	2%	15%	NA	NA	NA	NA
Lai, B. [31]	Trial-Based	2012	$	NA	NA	NA	NA	NA	NA	NA	NA	NA	NA
Blaikie, D.C. [20]	Model Based	1976	$	$13,578,891.38	$12,640,528.99	$938,362.38	Cost-effectiveness ratios	1.07	1.47	563,175	$6,179,117.15	$278,349.66	NA
O’Neill, C. [26]	Trial-Based	2014	£	$1601.31	$1271.45	$329.86	DMFS	2.6	3.9	253.7	$329.86	$2429.37	$2872.75
Stearns, S.C. [18]	Model Based	2006	$	$64.44	$336.01	(−) $271.58	No. of IMB visits	4	0	68	$39.55	NA	$40.96
Gibbs, L. [32]	Trial-Based	2012	$ AU	NA	NA	NA	Percentage of not having debris	56%	Referent	NA	NA	NA	$296,651.45
Donaldson, C. [25]	Trial-Based	1974	£	NA	NA	$346.01	DMFT	0.37	2.47	3.4	NA	NA	NA

ICER: incremental cost-effectiveness ratios, DMFT: decayed missing filled teeth, QALY: quality-adjusted life year, No. of IMB: number of “into the mouth of babes” program visits.

**Table A3:** The Drummond checklist for the risk of bias assessment of the trial-based economic evaluation studies.

Drummond Checklist/Study Authors	Anopa, Y. [19]	Blaikie, D.C. [20]	Kowash, M.B. [15]	Stearns, S.C. [18]	Samnaliev, M. [21]	Pukallus, M. [16]	Quinoez, R.B. [17]	Plonka, K.A. [23]	Folayan, M.O. [30]	Takeuchi, R. [22]
Was a Well-Defined Question Posed in an Answerable Form?	yes	yes	yes	yes	yes	yes	yes	yes	yes	yes
Was a Comprehensive Description of the Competing Alternatives Given?	yes	yes	yes	yes	yes	yes	yes	yes	yes	no
Was the Effectiveness of the Program Established?	yes	yes	yes	yes	yes	yes	no	yes	yes	yes
Were All the Important and Relevant Costs and Consequences for Each Alternative Identified?	yes	yes	yes	yes	yes	yes	yes	no	NA	no
Were Costs and Consequences Measured Accurately in Appropriate Physical Units?	yes	yes	yes	yes	yes	yes	yes	NA	NA	yes
Were Costs and Consequences Valued Credibly?	yes	yes	yes	yes	yes	yes	yes	NA	NA	yes
Were Costs and Consequences Adjusted for Differential Timing?	yes	yes	no	yes	NA	yes	yes	NA	NA	NA
Was an Incremental Analysis of Costs and Consequences of Alternatives Performed?	yes	yes	yes	yes	yes	yes	yes	NA	NA	NA
Was Allowance Made for Uncertainty in the Estimates of Costs and Consequences?	no	no	no	yes	NA	no	no	NA	NA	NA
Did the Presentation and Discussion of Study Results Include All Issues of Concern to Users?	yes	yes	yes	yes	yes	yes	yes	yes	yes	Yes
Score	9 from 10	9 from 10	7 form 10	10 from 10	8 from 10	9 from 10	8 from 10	4 from 10	4 from 10	5 from 10

**Table A4:** The Drummond checklist for the risk of bias assessment of the model-based economic evaluation studies.

Drummond Checklist/Study Authors	Donaldson, C. [25]	Davies, G.M. [29]	Koh R. [28]	Hietasalo P. [5]	O’Neill, C. [26]	Tickle, M. [24]	Reiss, M.L. [27]	Lai, B. [31]	Gibbs, L. [32]
Was a Well-Defined Question Posed in an Answerable Form?	yes	yes	yes	yes	yes	yes	yes	yes	yes
Was a Comprehensive Description of the Competing Alternatives Given?	yes	no	yes	yes	yes	yes	yes	yes	no
Was the Effectiveness of the Program Established?	yes	yes	yes	yes	no	no	yes	yes	yes
Were All the Important and Relevant Costs and Consequences for Each Alternative Identified?	no	no	yes	yes	yes	yes	no	NA	yes
Were Costs and Consequences Measured Accurately in Appropriate Physical Units?	yes	yes	yes	yes	yes	yes	yes	NA	yes
Were Costs and Consequences Valued Credibly?	yes	yes	yes	yes	yes	yes	yes	NA	yes
Were Costs and Consequences Adjusted for Differential Timing?	yes	yes	no	NA	yes	NA	No	NA	NA
Was an Incremental Analysis of Costs and Consequences of Alternatives Performed?	yes	no	yes	yes	yes	yes	No	NA	NA
Was Allowance Made for Uncertainty in the Estimates of Costs and Consequences?	yes	NA	yes	yes	yes	yes	NA	NA	NA
Did the Presentation and Discussion of Study Results Include All Issues of Concern to Users?	no	No	yes	yes	yes	yes	yes	yes	no
Score	8 from 10	5 from 10	9 from 10	9 from10	9 from 10	8 from 10	6 from 10	4 from 10	5 from 10
